# The Young Mother and the Fat Hog

**Published:** 1910-11

**Authors:** J. W. Hurty

**Affiliations:** Secretary, Indianapolis


### The Young Mother and The Fat Hog.

NOT A FABLE. SIMPLY STRAIGHT GOODS.

One time a little mother, who was only 25 years old, began to feel tired all the time. Her appetite had failed her for weeks before the tired feeling came. Her three little girls, once a joy in her life, now became a burden to her. It was, "mamma," "mamma," all day long. She never had noticed these appeals until the tired feeling came. The little mother also had red spots on her cheeks and a slight dry cough. One day, when dragging herself around, forcing her weary body to work, she felt a sharp but slight pain in her chest, her head grew dizzy, and suddenly her mouth filled with blood. The hemorrhage was not severe but it left her very weak. The doctor she had consulted for her cough and tired feeling had said, "You are all run down, you need a tonic." For a fee he prescribed bitters made of alcohol, water and gentian. This gave her false strength for a while, for it checked out her little reserve. When the hemorrhage occurred she and all her neighbors knew she had consumption, and the doctor should have known it and told her months before.

Now she wrote to the State Board of Health and said: "I am told that consumption in its early stages can be cured by outdoor life, continued rest and plenty of plain, good food. I do not want to die. I want to live and raise my children to make them good citizens. Where can I go to get well?" The reply was: "The great Christian State of Indiana has not yet risen to the mighty economy of saving the lives of little mothers from consumption. At present the only place where you can go is a grave. However, the State will care for your children in an orphans' asylum after you are dead, and then in a few years a special officer will find a home for them. But save your life—never." "That is a cranky idea," for a member on the floor of the Sixty-fifth Assembly said so. Besides, said he, "It isn't business; the State can't afford it." So the little mother died of the preventable and curable disease, the home was broken up and the children were taken to the orphans' asylum.

A big fat hog one morning found he had a pain in his belly. He squealed loudly and the farmer came out of his house to see what was the matter. "He's got the hog cholry," said the hired man. So the farmer telegraphed to Secretary Wilson of the United States Agricultural Department (who said the other day he had 3000 experts in animal and plant diseases), and the reply was: "Cert., I'll send you a man right away." Sure enough, the man came. He said he was a D. V. S. and he was, too. He had a government syringe and a bottle of government medicine in his hand bag, and he went for the hog. It got well. It wasn't cranky for the government to do this, and it could afford the expense, for the hog could be turned into ham, sausage, lard and bacon.

Anybody, even a fool, can see it would be cranky for the State to save the life of a little mother, and it could not afford it either.

Moral: Be a hog and be worth saving.—*Bulletin Indiana State Board of Health, Dr. J. W. Hurty, Secretary,* Indianapolis.

